# Maintaining helper wellness and competence in a shared trauma reality

**DOI:** 10.1186/s13584-016-0102-7

**Published:** 2016-09-06

**Authors:** James Halpern

**Affiliations:** Institute for Disaster Mental Health, State University of New York at New Paltz, 600 Hawk Drive, New Paltz, NY 12561 USA

## Abstract

As shared trauma reality becomes more common in Israel and other countries, this commentary argues that we need more research to inform how to best assist mental health professionals who are both victims and helpers in the aftermath of traumatic events. Typical remedies for the occupational hazards of working with trauma survivors may not apply for those who are exposed to a prolonged terror threat. Research-informed self-care strategies need to be identified and practiced.

## Commentary

In a timely paper on the effects of shared war reality on the professional quality of life (ProQol) in mental health professionals (MHPs), Peruginin and his colleagues [1] review the impact of shared trauma on MHPs in Southern Israel. MHPs in Otef Gaza, exposed to constant threat and frequent missile attacks, are both directly and vicariously traumatized. Studies cited by the authors show these MHPs to have higher rates of psychological and family problems and also the positive outcomes of compassion satisfaction and vicarious posttraumatic growth. As disasters and complex emergencies become larger in scope, intensity and duration and shared trauma reality becomes more common, this area of research is critical in promoting the health and wellbeing of first and second responders. In the US, some MHPs lost friends and relatives to the 9/11 attacks, while others, assisting Hurricane Katrina survivors, lost their own homes and valued possessions. In the US and in Europe counselors working with survivors of terrorism and or gun violence have been victims themselves and in some neighborhoods feel under chronic and constant threat. How do we best identify and support MHPs who are helpers as well as victims sharing trauma with the population they serve? Unfortunately this is an all too common experience for MHPs in Southern Israel. Frequently it is necessary for MHPs to take shelter alongside a client, when a siren warns of an incoming missile in the middle of a counseling session. This paper begins to explore the experience of shared war reality in and beyond Otef Gaza.

The occupational hazards of burnout, secondary traumatic stress and compassion fatigue associated with doing trauma work are common and dangerous. MHPs can lose effectiveness, or can be lost to substance abuse or leave the profession entirely. Estimates suggest that between 40 % and 85 % of “helping professionals” develop some aspect of these hazards [2] and the International Society of Traumatic Stress Studies (ISTSS) acknowledges that ‘anyone who encounters trauma survivors empathically and is committed to helping them’ may be vulnerable to this array of occupational hazards. Are these occupational hazards more significant for helpers who themselves are under threat? The results of the present study suggest that many helpers from Otef Gaza and Beer Sheva have found ways to successfully cope with shared war reality. A better understanding of how they accomplished this could benefit helpers around the world.

Studies of civilians living with constant terror are rare, but one conducted after the second Palestinian Intifada showed that every citizen in Otef Gaza was either directly or indirectly exposed to terror events and that all struggled to cope with ongoing uncertainty [3]. Civilians under constant attack experience a unique type of trauma where many of the typical risk and resilience factors do not apply. The “dose–response relationship,” meaning the bigger the dose of traumatic event a person experiences, the worse his or her psychological reaction, is well established [4]. But, this “more = worse” finding does not hold up with prolonged terrorist threats. Studies done in the South of Israel after the 2008/2009 conflict [5], and the 2014 Israel-Hamas war [6], showed no connection between trauma symptoms and proximity to the Gaza Strip. Both studies looked at traumatic exposure according to the number of seconds it took for residents to take shelter from an incoming missile in response to an air-raid siren. Those with 60 or 90 s to find shelter showed no difference in trauma symptom scores from those with only 15 s to escape. Similar to the findings on civilians, Peruginin and colleagues found no difference in ProQol scores between the more highly exposed group of MHPs from Otef Gaza (15 s to reach shelter) and the MHPs from Beer Sheva (60 s to reach shelter). This may be due to the fact that the difference in seconds to find shelter does not mitigate the fact that all are sufficiently exposed and under threat. How far away do civilians and MHPs need to be from attack in order to feel safe? This question has important health policy implications for survivors and helpers. If conflict along the Gaza Strip is anticipated, the results of the paper suggest that residents and MHPs may need to be moved far enough away to be out of missile range to feel safe. The results also suggest that these MHPs close to danger have found ways to cope.

The authors speculate that the no difference could be due to the MHPs from Otef Gaza being better prepared, better trained and having a greater sense of community-belonging, solidarity and trust in their leaders. Resilience Centers may have provided more training and better tools to help their clients. This is an important area of research to explore. If MHPs are better trained to care for others and feel more confident in their ability to offer effective services, however close to the traumatic event, are they more resilient? And if so, what kinds of professional trainings are the most effective and protective?

The paper also points out that MHPs in Otef Gaza, as a result of their exposure, may have learned new and better coping mechanisms, suggesting that uniquely specialized coping skills are essential for those exposed to continuous threats. One of the most research supported effective ways for survivors and helpers to cope is to access social support [7]. However, wartime conditions, unlike other traumas, create stress on the entire population limiting the usefulness of social support. Survivors looking for external support may be disappointed to discover that friends and family are in the same situation and less capable of offering support [5, 8]. Have MHPs in Otef Gaza or Beer Sheva learned to be less reliant on social support? Can we identify what coping skills are most effective for those experiencing shared trauma to minimize compassion fatigue and maximize compassion satisfaction? MHPs in the South of Israel and in other countries need research informed guidance on how to best cope with these challenges. One project funded by USAID brought together MHPs from Israel, West Bank, Gaza and the US to develop psychoeducational materials for victims of violence as a well as to support caregivers and helpers. Psychoeducational materials addressing the self-care needs of helpers in Hebrew, Arabic, Russian and English were developed and widely disseminated throughout the region [9]. More research is needed to determine if these materials or additional trainings or courses can help helpers to cope with the increasing likelihood of shared war/trauma reality. The authors claim that additional trainings, courses or preparedness is helpful to MHPs wellbeing needs to be validated through further research.

What may be most notable about the results of this study is not just the no difference in ProQol scores for MHPs in Beer Sheva and Otef Gaza, but that the scores are lower than one might expect in both communities. Serving others during times of extreme crisis shapes helpers, often in positive and satisfying ways. MHPs who have responded to catastrophic events have reported rewards including: immediate and gratifying personal satisfaction from helping others, feelings of empowerment during times of potentially debilitating crisis, relief from routine mental health work, emotional bonding with responder teams and community, and a sense of privilege resulting from providing mental health services in unique circumstances when they’re sorely needed [4]. An often-unanticipated reward that many disaster/emergency responders identify is how the professional ‘lessons learned’ can also positively impact their personal lives. Although much has been written about the dangers of work life spilling over into personal/family life, this effect has potentially positive benefits as well. Responders have reported feeling greater empathy and compassion for and with family members and friends, a greater awareness of what is important in life, appreciation for the value of human connection, tolerance for difference, and profound gratitude for the opportunities and relationships they have in their lives. The occupational hazards and rewards of the work can occur simultaneously. The distress of the work does not preclude growth and, in fact, is often conducive to growth. Trauma and disaster work changes the way we think – both positively and negatively. Vicarious posttraumatic growth is most likely to occur if MHPs are exposed to the growth of those they are assisting [10]. The ProQol scores in MHPs from Beer Sheva and Otef Gaza may be due to the commitment, strength, resilience and growth in the populations they are serving. Perhaps we need to credit the clients, the civilian population in Southern Israel, for the wellbeing of the MHPs as much as supervisors and trainers.

What do MHPs, exposed to shared war/trauma reality, need to prevent or recover from the occupational hazards and even to grow and flourish in this work? Effective counseling requires rapport, attachment and connection between client and helper. MHPs who are exposed to the same traumatic events as their clients might be seen as more understanding and empathic, more appreciated and more successful. What are the risk and resilience factors for helpers who are directly exposed to the traumatic events? Research on shared war reality needs to focus on possible risk and resilience factors such as:

- The personality of the helper (e.g. hardy, optimistic, politically committed)
- View of professional self as competent and purposeful
- Perceived social support
- Absence of negative social support
- Training in evidence based trauma therapies
- Workshops on self-care
- Effective self care strategies and activity
- Years of experience and training
- Effective supervision
- Organization commitment to the well being of helpers.

The authors suggest that qualitative research is needed and this is an important starting place. We could begin by simply asking MHPs in the region, who are successful and resilient, why they believe they are doing so or what kinds or services or support they need, or what helped them most during previous crises.

A commitment to the wellbeing of MHPs serving communities under threat should be a priority for public health leaders and MHPs themselves. Helpers need the time for proper preparation and self-care. If those in authority ignore the care of helpers, or if MHPs don’t care for themselves, the unmanaged distress may not only adversely affect the wellbeing of helpers, but potentially that of clients and the response effort as a whole. As a result, commitment to the wellbeing of MHPs and their practice of good self-care to maintain personal welfare is not a luxury, but an ethical responsibility. Many physical and psychosocial stress-related problems can be prevented through regular self-care practices. It is imperative to find more effective ways of encouraging good self-care beyond admonishing MHPs to take better care of themselves. MHPs need to commit to integrating self-care into their daily practices; think about how they can flourish rather than survive as counsellors and always consider the reciprocal relationship between care of self and care of others [11]. Rabbi Hillel understood well over 2000 years ago that care for others is intertwined with care for the self: “If I am not for myself, who will be for me? If I am only for myself, what am I? And, if not now, when?”
